# CLSI Validation of Exchangeable Copper Determination in Serum by ICP-MS: A Focus on Alzheimer’s Disease and Wilson Disease

**DOI:** 10.3390/biom15060788

**Published:** 2025-05-29

**Authors:** Rosanna Squitti, Amit Pal, Irena D. Ivanova, Massimo Marianetti, Mauro Rongioletti

**Affiliations:** 1 Department of Laboratory Science, Research and Development Division, Ospedale Isola Tiberina—Gemelli Isola, 00186 Rome, Italy; maurociroantonio.rongioletti@fbf-isola.it; 2 Department of Theoretical and Applied Sciences, eCampus University, Viale Massenzio Masia, 26, 22100 Como, Italy; 3 Department of Biochemistry, All India Institute of Medical Sciences, Kalyani 741245, West Bengal, India; amit.biochem@aiimskalyani.edu.in; 4 Department of Clinical Laboratory, St. Ivan Rilski University Hospital, Medical University, 1431 Sofia, Bulgaria; irena.dimitrova@gmail.com; 5 Department of Neuroscience and the Experimental Alzheimer Center, Fatebenefratelli Roman Province, Via Cassia 600, 00189 Rome, Italy; marianetti.massimo@fbfgz.it

**Keywords:** diagnostic biomarker, exchangeable copper, sensitivity, specificity, ICP-MS

## Abstract

Background: Copper dyshomeostasis has been implicated in a subset of Alzheimer’s disease (AD) patients, characterized by elevated non-ceruloplasmin-bound copper (non-Cp Cu). However, traditional methods for estimating non-Cp Cu are indirect and analytically imprecise. This study introduces and validates a direct assay for exchangeable copper (ExcCu) by inductively coupled plasma-mass spectrometry (ICP-MS), compliant with Clinical and Laboratory Standards Institute (CLSI) guidelines. Methods: We performed analytical validation of the ExcCu assay following CLSI protocols (EP5, EP6, EP7, EP9, EP15, and EP28). ExcCu and other copper-related biomarkers were quantified in serum samples from 154 healthy controls, 82 AD patients, and 10 patients with Wilson disease (WD). Diagnostic performance was evaluated via receiver operating characteristic (ROC) curve analysis, and inter-method agreement was assessed using Bland–Altman plots. Results: The ExcCu assay demonstrated excellent linearity, precision (CV < 6%), and inter-laboratory reproducibility. Among AD patients, ExcCu levels were significantly elevated compared to controls (*p* < 0.001). ExcCu distinguished AD from controls with an AUC of 0.80 and a specificity of 95%. Compared to non-Cp Cu, ExcCu yielded no negative values and showed reduced bias. The relative exchangeable copper (REC) index was more effective in differentiating AD from WD (AUC = 0.88). Conclusions: The validated ExcCu assay overcomes the limitations of the traditional non-Cp Cu calculation, offering a reliable biomarker for copper-related AD subtypes. Its high specificity supports its use in patient stratification, potentially contributing to personalized approaches in AD diagnosis and therapy.

## 1. Introduction

According to the World Alzheimer Report 2021, more than 55 million people are currently living with dementia worldwide, with nearly 10 million new cases each year. Alzheimer’s disease (AD) is the most common form and is expected to affect over 150 million people by 2050. In recent years, several anti-amyloid monoclonal antibodies have been developed to slow disease progression. Aducanumab received accelerated FDA approval in 2021, although its clinical efficacy remains controversial. In contrast, Lecanemab—approved by the FDA in 2023 and by the EMA in 2024—demonstrated a 27% reduction in cognitive decline over 18 months in patients with early-stage AD [1]. Among the available anti-amyloid agents, Donanemab has shown the strongest clinical effect [2], while Gantenerumab, despite achieving amyloid clearance, failed to meet its primary efficacy endpoints [3]. Notably, Lecanemab’s efficacy appeared to be sex-dependent, with greater benefit observed in males (~43%) than in females (~12%) [4].

Despite these developments, none of these therapies represents a definitive cure. Their limited efficacy and potential side effects—such as amyloid-related imaging abnormalities (ARIA), including cerebral edema and microhemorrhages [5]—underscore the need to explore alternative pathogenic pathways. This is exemplified by the rejection of Donanemab’s approval in Europe, where regulatory authorities concluded that the therapeutic benefits did not outweigh the risks, primarily due to the high incidence of ARIA.

Among the non-amyloid-centric hypotheses, copper dyshomeostasis has emerged as a plausible contributor to AD pathogenesis. Non-ceruloplasmin-bound copper (non-Cp Cu), also known as “free copper”, refers to the fraction of serum or plasma copper not tightly bound to ceruloplasmin. Under physiological conditions, approximately 85–95% of circulating copper is stably bound to ceruloplasmin, while 5–15% exists as non-Cp Cu, loosely associated with albumin and amino acids (e.g., histidine), or as low-molecular-weight complexes [6]. This labile, redox-active pool can cross the blood–brain barrier and participate in pathological redox reactions. In Wilson disease (WD), defective ATP7B-mediated hepatic copper clearance leads to systemic copper overload and markedly increased non-Cp Cu levels, sometimes exceeding 50–100% of total serum copper [7]. These elevations are associated with oxidative stress, tissue toxicity, and neurodegeneration [8].

While the amyloid-centric-hypotheses framework acknowledges the role of amyloid-β (Aβ) aggregation, it proposes that aberrant copper metabolism—particularly elevated levels of non-Cp Cu—may act as an upstream driver by promoting oxidative stress and facilitating Aβ precipitation rather than placing amyloid at the core of the disease process [9]. Independent reviews and meta-analyses have confirmed a pattern of peripheral copper excess combined with cerebral copper deficiency in AD [10,11], supporting the notion that non-Cp Cu may represent a biomarker for a copper-related AD subtype. Genetic studies have also associated *ATP7B* variants—the gene responsible for WD—with disrupted copper regulation in AD patients [10,12,13,14]. Furthermore, the recently described mechanism of cuproptosis, a regulated form of copper-dependent cell death [15], provides a potential molecular link between elevated non-protein-bound copper levels and *ATP7B* dysfunction, strengthening the rationale for copper-targeted investigation in AD. However, mainstream methods for measuring non-Cp Cu are indirect and error-prone, relying on separate quantification of total copper and ceruloplasmin in serum. This approach (Walshe’s formula—provided in Section 2) often produces negative or inconsistent values [16]. In response, direct assays have been proposed, including a fluorescent probe developed in 2017 [17]. Although promising, this method was limited by rapid signal decay during transport, restricting its use to the site of synthesis and preventing its commercialization as a stable diagnostic kit.

To address these limitations, we developed and validated a direct ICP-MS-based method for measuring exchangeable copper (ExcCu). This study assesses its analytical robustness and clinical relevance in comparison to traditional non-Cp Cu estimation, with the goal of establishing a reliable and clinically applicable biomarker for copper-related AD phenotypes.

## 2. Methods

### 2.1. Subjects

Serum samples from 154 cognitively healthy individuals were provided by the Blood Donor Unit of Fatebenefratelli Hospital (Isola Tiberina, Rome, Italy). All participants were screened to exclude any history or clinical evidence of neurological, psychiatric, or cardio-cerebrovascular disease. The presence of cognitive impairment was excluded using the Mini-Mental State Examination (MMSE) [18] and the Montreal Cognitive Assessment (MOCA) [19]. Serum samples were used for two separate analytical procedures: non-Cp Cu was estimated in the Rome laboratory, while ExcCu was quantified at IGEA Research Corporation (Miami, FL, USA). The data from the healthy control group were newly collected and have not been previously published.

Eighty-two patients diagnosed with mild AD were enrolled between January 2018 and July 2021 from the Department of Neuroscience and the Experimental Alzheimer Center, Fatebenefratelli Roman Province, both located in Rome. Diagnostic procedures for AD followed established clinical criteria, as previously described [20]. Exclusion criteria included abnormal thyroid, liver, renal, or cardiac function, as well as a history of stroke, focal neurological signs, significant white matter pathology, or evidence of hemodynamically relevant stenosis or the occlusion of cervical or intracranial vessels.

Additionally, 10 patients with WD were recruited at the time of diagnostic assessment at St. Ivan Rilski University Hospital, Medical University of Sofia (Sofia, Bulgaria). Diagnosis was established according to current guidelines, including the Leipzig criteria, and confirmed by genetic analysis [21].

Venous blood samples were drawn after overnight fasting and collected into trace-element-free tubes (e.g., Vacutainer^®^ BD Thrombin, Franklin Lakes, NJ, USA). Sera were separated by centrifugation (3000 rpm, 10 min, 0 °C), aliquoted (0.5 mL), and rapidly stored at −80 °C until analysis. A portion of each serum sample was shipped under frozen conditions to IGEA Research Corporation (Miami, FL, USA) for ExcCu analysis, and to the Biology Laboratory, Ospedale Isola Tiberina-Gemelli Isola (Rome, Italy), for the measurement of total copper, ceruloplasmin concentration, and ceruloplasmin enzymatic activity.

All experimental procedures involving human subjects were conducted in compliance with institutional ethical standards following the Declaration of Helsinki (1975, revised 2008). The study protocol was reviewed and approved by the local institutional ethics committee (approval number 1/1991; 26/2014), and all participants provided written informed consent under applicable regulations. All sample analyses were performed in a blinded fashion, and materials certified to be free of trace metal contamination were used throughout the workflow.

### 2.2. Analytical Methods

The analytical procedure for ExcCu determination was developed and optimized at IGEA Research Corporation (Miami, FL, USA) based on published protocols with minor modifications [22,23,24]. Following El Balkhi et al. (2011) [23], this method quantifies exchangeable copper—the labile fraction mobilized by EDTA incubation—rather than ultrafiltrable copper. While we employed a 100 kDa filter for serum ultrafiltration instead of the 30 kDa filter used by El Balkhi [22,23], both retain albumin and larger copper-binding proteins, ensuring methodological equivalence in the operational definition of ExcCu [22,23,25].

### 2.3. Reagents and Solutions

The following reagents were used: TraceMetal Grade Nitric Acid (Fisher Scientific, A509-P500); EDTA-disodium salt dihydrate (Sigma, E5134-50G, New York, NY, USA); copper standard, 1000 µg/mL (SPEX CertiPrep, CLCU2-2M, Metuchen, NJ, USA); yttrium internal standard, 10 µg/mL (SPEX CertiPrep, CLY2-1AM); and Type I distilled water (DW-1). The solutions were prepared as follows: EDTA solution (3 g/L, 8 mM); 2% nitric acid; 2% nitric acid with yttrium; and copper standards (serial dilution), prepared from 10,000 µg/mL stock by successive 1:10 dilutions to obtain 10^4^, 10^3^, and 10^2^ µg/L. To validate the measurements and as a standard for copper serum analysis, pooled healthy serum reference samples, SeronormTM Trace Elements Serum L-1 and L-2 (SERO AS, Lysaker, Norway), were purchased.

### 2.4. Assay

Copper and ExcCu in serum were quantified using an iCAP Q ICP-MS (ThermoFisher, Waltham, MA, USA) calibrated for Cu-65 over the range 0.1–10.0 µg/dL (0.0157–1.57 µmol/L). All procedures were performed under standard lab safety conditions. For ExcCu, 100 µL of serum was mixed with 100 µL of 3 g/L EDTA solution and incubated for 1 h at room temperature. For ultrafiltration, 400 µL of diluted EDTA solution was added. Next, 500 µL of this mixture was loaded into a 100 kD centrifugal filter unit (Amicon Ultra, Millipore Sigma, Burlington, MA, USA-0.5 mL) and centrifuged at 7000× *g* for 30 min at room temperature. All filters were pre-rinsed with 2% nitric acid followed by ultrapure water to remove any trace metal residue. Dilution for ICP-MS: 300 µL of filtrate was mixed with 900 µL of 2% nitric acid containing 100 µg/L yttrium (internal standard), for a final dilution factor of 40. For copper assessment, 100 µL of serum was mixed with 400 µL of water and 1000 µL of 2% nitric acid containing 0.1 µg/mL of yttrium (as an internal standard). Controls and calibration: A background control used 300 µL of water instead of filtrate. Standard curve samples were prepared by spiking background controls with known concentrations of copper (1, 10, and 100 µg/L). Measurement and quality control: Each sample was analyzed in triplicate. The results were averaged. Parallel sample duplicates were also analyzed; results were accepted only if the variation was <10%. Otherwise, the test was repeated. The ICP-MS settings for the copper quantification are reported in Supplementary Table S1.

### 2.5. Biochemical and Molecular Investigations: Standard Copper Studies

The panel of copper-related biomarkers—namely, serum copper, ceruloplasmin concentration, ceruloplasmin enzymatic activity, and non-Cp Cu—was measured using standardized methods as described previously [20].

To compute the non-Cp Cu index, ceruloplasmin levels were measured through an immunoassay-based method, and the calculation followed the approach originally proposed by Walshe [16] and known as “Walshe’s formula”. For each individual, the concentration of copper bound to ceruloplasmin (CB) was calculated as:

CB (µmol/L) = Cp (mg/dL) × 10 × *n*

where *n* = 0.0472 µmol/mg, derived from the atomic mass of copper (63.546 g/mol) and the molecular weight of ceruloplasmin (~132,000 Da).

Subsequently, non-Cp Cu was obtained by subtracting the CB from the total serum copper:

non-Cp Cu (µmol/L) = Total serum copper (µmol/L) − CB (µmol/L)

This method yields values in micromolars (µmol/L) and is grounded in the assumption that each ceruloplasmin molecule binds approximately 6–8 copper atoms. Reference values for non-Cp Cu are generally considered within the 0–1.6 µmol/L range in healthy individuals [8,16].

Additionally, the copper-to-ceruloplasmin ratio (Cu:Cp) was used as an internal quality control index to assess the stoichiometric balance between copper and its carrier protein. Following the method proposed by Twomey et al. [26,27,28], the ratio was calculated as:

Cu:Cp = [Cp (mg/dL)] × 10^4^ [Copper (µmol/L)] × 132,000

In physiologically normal conditions, a Cu:Cp ratio close to 6.6 is considered representative of optimal copper incorporation into ceruloplasmin [26,27,28].

Finally, the relative exchangeable copper (REC) calculation index was calculated as the ratio of exchangeable copper (ExcCu, µmol/L) to total serum copper (µmol/L), expressed as a percentage:

REC (%) = (Total ExcCu) × 100

This index has been proposed as a reliable biomarker for WD diagnosis [29,30,31].

### 2.6. Statistical Methods

Data were analyzed using SPSS (version 29.0, IBM SPSS Statistics for Windows) and Python (v3.10) with SciPy, statsmodels, and scikit-learn libraries. Descriptive statistics were computed for demographic and biochemical variables across diagnostic groups (healthy controls, AD, and WD). Continuous variables were expressed as the mean ± standard deviation (SD), and categorical variables as counts and percentages.

Group comparisons were performed using one-way ANOVA, followed by Tukey’s HSD post hoc tests. Correlations between copper-related biomarkers (e.g., non-Cp Cu, ExcCu) and demographic or biochemical parameters (e.g., age, sex, ceruloplasmin) were assessed using Pearson’s correlation coefficient (r).

Reference intervals for ExcCu in healthy controls were calculated following the Clinical and Laboratory Standards Institute (CLSI) C28-A3 guidelines [32], using non-parametric methods with 90% confidence intervals.

ROC curves were constructed to assess the diagnostic performance of ExcCu and non-Cp Cu in distinguishing AD and WD from healthy controls. AUC values were computed with 95% confidence intervals, and optimal thresholds were determined using the Youden Index [33]. From these, sensitivity, specificity, and positive and negative likelihood ratios (LR^+^, LR^−^) were calculated.

Agreement between ExcCu and non-Cp Cu was assessed via Bland–Altman analysis [34], both including and excluding negative values of non-Cp Cu to evaluate systematic bias due to the formula-based estimation.

All statistical tests were two-tailed, and *p*-values < 0.05 were considered statistically significant.

### 2.7. Analytical Validation of Exchangeable Copper (ExcCu) According to CLSI Guidelines

The ExcCu assay was validated following guidelines from the CLSI, using the StatisPro software (v3.02.2) to ensure full compliance. The following CLSI documents were applied:

EP05-A3: Evaluation of Precision Performance of Quantitative Measurement Methods; EP06-A: Evaluation of Linearity of Quantitative Measurement Procedures; EP17-A2: Protocols for Determination of Detection Capability (LoB, LoD); EP09c: Method Comparison and Bias Estimation Using Patient Samples; EP28-A3c: Defining, Establishing, and Verifying Reference Intervals in the Clinical Laboratory; EP24-A2: Assessment of Diagnostic Accuracy Using ROC Analysis; and Recovery Tests: Evaluation of analytical accuracy via spiking experiments.

Details of the experimental procedures and resulting performance metrics are provided in Section 3, in alignment with the CLSI validation framework.

## 3. Results

**Figure 1:**
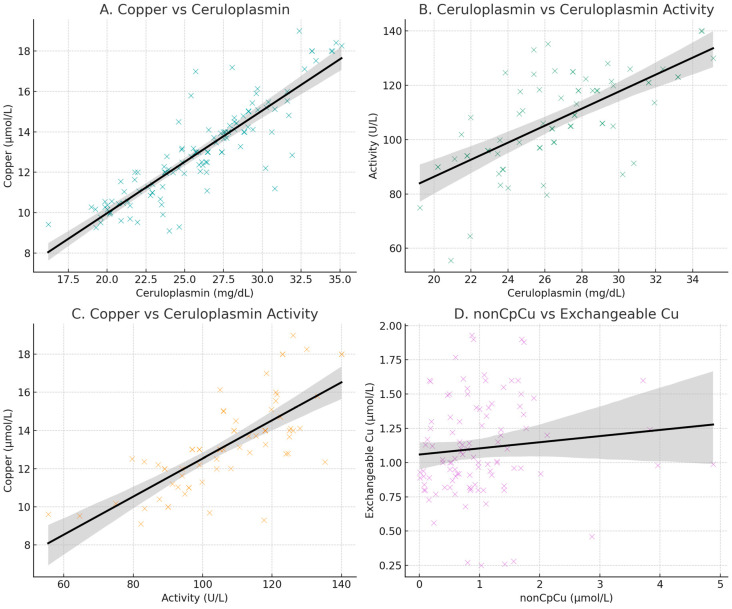
Scatter plots of copper and ceruloplasmin concentration (**A**); ceruloplasmin concentrations and ceruloplasmin activity (**B**); copper and ceruloplasmin activity (**C**); scatter plot of values of non-ceruloplasmin-bound copper (non-Cp Cu; indirect method) and exchangeable copper (ExcCu; direct method) (**D**).

A total of 154 healthy controls (69 women, 45%), mean ± SD age 58.8 ± 17.4 years old (20–90), 10 WD subjects (8 women, 80%), mean age 44 ± 13.7 years old (32–65), and 82 AD patients (46 women, 56%), mean age 70.45 ± 7.8 (52–88) were recruited. In healthy controls, the correlation between ceruloplasmin concentrations and serum copper levels was 0.88 (*p* < 0.0001), the correlation between ceruloplasmin concentrations and ceruloplasmin activity values was 0.66 (*p* < 0.0001), and that between ceruloplasmin activity values and serum copper levels was 0.74 (*p* < 0.0001) and non-Cp Cu vs. ExcCu (r = 0.10; *p* = 0.290) (Figure 1).

The Cu:Cp ratio in healthy controls was 6.63 (0.55), demonstrating a good accordance between copper and ceruloplasmin, allowing the application of Walshe’s formula.

### 3.1. Exchangeable Copper Test CLSI Validation

Procedures for CLSI validation were applied for the ExcCu method. A description of the CLSI procedures is detailed in Section 3, for a better description. The “Evaluation of the Linearity of Quantitative Measurement Procedures: A Statistical Approach; Approved Guideline” from the Clinical and Laboratory Standards Institute (CLSI EP06-A guideline) was applied.

### 3.2. Precision (EP05-A3)

Precision was assessed over 20 non-consecutive days, with two runs per day and two replicates per run (20 × 2 × 2 design). Serum pools with low (0.86 µmol/L), medium (1.21 µmol/L), and high (2.08 µmol/L) ExcCu concentrations were analyzed. Coefficients of variation (CVs) for repeatability ranged from 2.7% to 4.1%, while within-laboratory CVs ranged from 4.4% to 6.8% (Table 1). A detailed component analysis of variance is provided for each concentration level: low (Table 1A), medium (Table 1B), and high (Table 1C).

### 3.3. Linearity (EP06-A)

**Figure 2:**
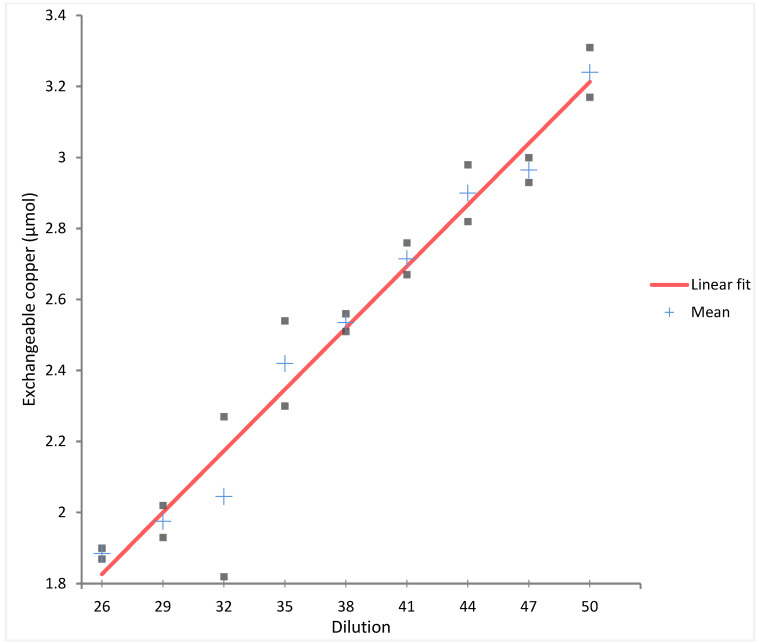
Linearity plot of exchangeable copper (ExcCu) across the dilution range.

Nine concentration levels were tested in duplicate. Linear regression showed strong linearity with no statistical improvement from second or third-order polynomial models. Figure 2 shows the linearity plot across the dilution range.

Supplementary Table S2 reports the target concentration levels used to assess the linearity and accuracy of ExcCu measurement. These levels were obtained by spiking known amounts of copper into the assay background matrix. For each level, triplicate ICP-MS measurements were performed, and the observed values were compared to the expected concentrations to evaluate recovery and model fit. The results confirm the assay’s ability to reliably quantify ExcCu across a clinically relevant concentration range.

The linearity of the ExcCu assay was evaluated using weighted regression models of increasing complexity (linear, second-, and third-order polynomial fits). Table 2 reports the estimated regression parameters, including the intercept (Constant), slopes (X, X^2^, X^3^), their corresponding standard errors (SE), t-statistics, degrees of freedom (DF), and *p*-values for significance testing. Root Mean Square Error (RMSE) values are also provided as a measure of model fit.

### 3.4. Limit of Detection (EP17-A)

A total of 60 measurements (12 replications on 5 samples) on blanks and 60 measurements (12 replications on 5 samples) on low-level samples were carried out. With α and β set at 5%, the limits were determined as follows:

- LoB (Limit of Blank): 0.0002 µmol/L
- LoD (Limit of Detection): 0.0397 µmol/L

Values are reported in Table 3.

### 3.5. Recovery

The recovery of ExcCu was evaluated using standard serum samples spiked with known concentrations ranging from 0.5 to 5.0 µmol/L. Each level (0.5, 1.0, 2.0, 3.0, and 5.0 µmol/L) was tested in nine replicates and compared to a control sample spiked with water only. The recovery rates ranged from 94.6% to 96.0%, with a median value of 95.0%, demonstrating the high accuracy of the assay across the tested range. Detailed recovery data are provided in Supplementary Table S3.

### 3.6. Agreement and Concordance Analysis

**Figure 3:**
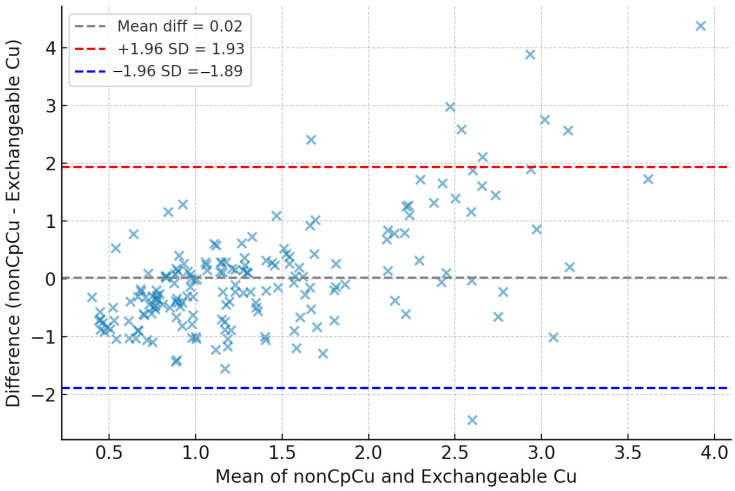
Bland–Altman analysis comparing non-ceruloplasmin-bound copper (non-Cp Cu) and exchangeable copper (ExcCu) after excluding physiologically implausible negative values of non-Cp Cu.

A Bland–Altman analysis comparing non-Cp Cu and ExcCu revealed a mean bias of −0.28 µmol/L, with wide limits of agreement (−2.16 to +2.72 µmol/L). A Bland–Altman analysis excluding negative values of non-Cp Cu revealed no systematic bias between non-Cp Cu and ExcCu (mean difference = +0.02 µmol/L; limits of agreement: −1.89 to +1.93 µmol/L). Despite this, the correlation between the two methods was only moderate (r = 0.46, *p* < 0.0001), indicating that they are not fully interchangeable (Figure 3).

The reference interval for ExcCu was calculated in the control group using a parametric method (mean ± 1.96 * standard deviation). The analysis revealed a reference interval for serum ExcCu in healthy controls (n = 154) of 0.27 to 1.90 µmol/L, with 90% confidence intervals of 0.27–0.44 µmol/L for the lower limit and 1.63–1.93 µmol/L for the upper limit. This interval provides a baseline for evaluating ExcCu levels in healthy individuals and can be used as a comparative benchmark when analyzing patient data.

These results highlight the variability in ExcCu concentrations among controls and support the use of this interval in distinguishing normal from pathological values. The correlation between ExcCu and both sex and age was evaluated in the control group. ExcCu did not correlate with sex or age (*p* > 0.2), while non-Cp Cu was slightly increased in women (r = 0.28, *p* = 0.004) but did not correlate with age in the current dataset (*p* > 0.2).

These results indicate that sex may play a role in determining ExcCu levels, while age does not appear to be a significant factor. The upper reference limit (95%) for ExcCu was 1.9 µmol/L (CI 90%: 1.63 to 1.93).

**Figure 4:**
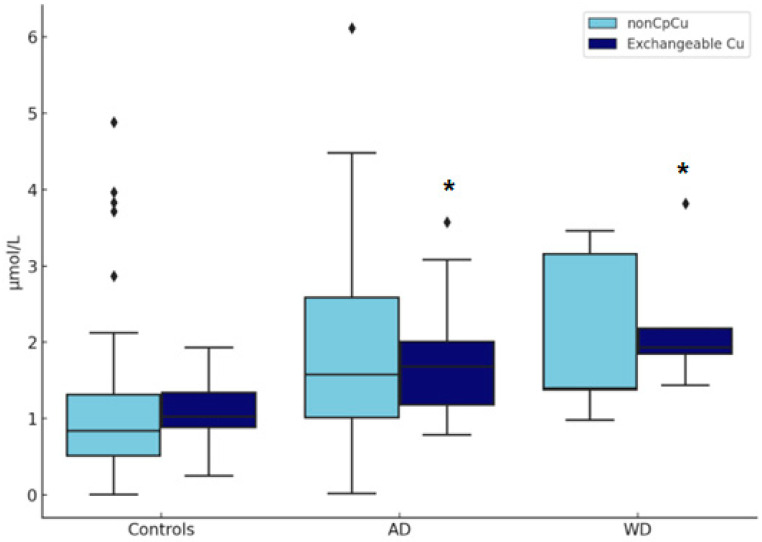
Comparing non-ceruloplasmin-bound copper (non-Cp Cu) and exchangeable copper (ExcCu) mean values for healthy, Alzheimer’s disease, and Wilson disease individuals * *p* < 0.001.

As shown in Figure 4, a one-way ANOVA revealed significant group differences in ExcCu levels [F(2,244) = 45.62, *p* < 0.001)], while non-Cp Cu levels showed a trend toward significance [F(2,209) = 10.85, *p* < 0.001].

**Figure 5:**
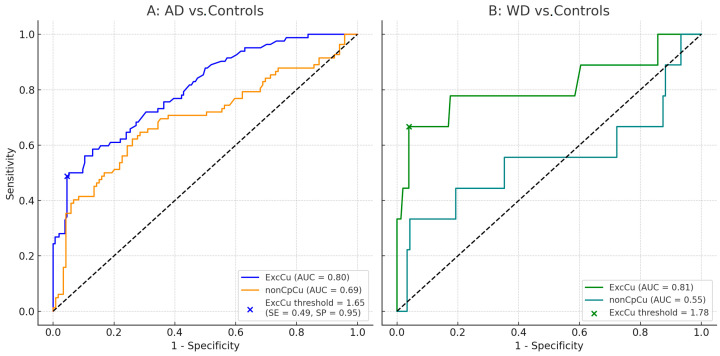
ROC curves of the comparison between exchangeable copper (ExcCu; AUC = 0.80) (blue line) and non-ceruloplasmin-bound copper (non-Cp Cu; AUC = 0.69) (orange line) in discriminating healthy controls vs. Alzheimer’s disease (AD) patients (**A**); x indicates the threshold for ExcCu in AD equal to 1.65. ROC curves of the comparison between ExcCu (AUC = 0.81) (green line) and non-Cp Cu (AUC = 0.55) (light blue line) in discriminating healthy controls vs. Wilson disease (WD) patients (**B**).

Post hoc Tukey’s tests indicated that ExcCu was significantly higher in AD patients compared to controls (mean difference = +0.58 µmol/L, *p* < 0.001) and even higher in WD patients (mean difference = +0.79 µmol/L, *p* < 0.001). The difference between WD and AD patients was not statistically significant (mean difference = +0.22 µmol/L, *p* = 0.4). Similarly, non-Cp Cu was higher in AD vs. healthy controls (+0.88, *p* < 0.001) and also in WD vs. healthy, but did not reach the statistical threshold (+1.07, *p* = 0.0551). Furthermore, non-Cp Cu was not different in AD vs. WD. In comparing AD patients and controls, ExcCu showed a higher discriminative performance (AUC = 0.80) than non-Cp Cu (AUC = 0.69). The optimal threshold for ExcCu was 1.65 µmol/L, yielding a specificity of 95% and a sensitivity of 49% (Figure 5A).

When comparing WD patients to controls, ExcCu again outperformed non-Cp Cu, with AUCs of 0.81 and 0.55, respectively (Figure 5B). For the discrimination between AD patients and controls, the optimal threshold of 1.65 µmol/L (SP 95.5%, 95% CI 92.2–98.8%, and SE 48.8%; CI 95%: 38.0–59.6%) for ExcCu yielded a positive likelihood ratio (LR+) of 10.84, indicating strong rule-in diagnostic value, and a negative likelihood ratio (LR−) of 0.54, suggesting limited rule-out capability.

**Figure 6:**
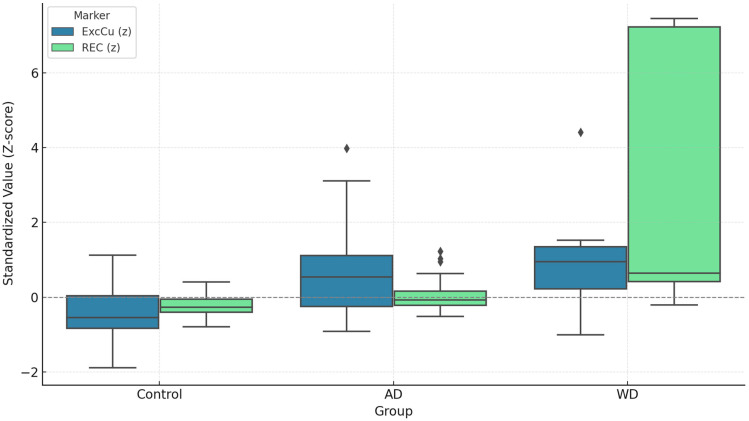
Standardized distributions of exchangeable copper (ExcCu) and relative exchangeable copper (REC) across diagnostic groups (control, Alzheimer’s disease [AD], and Wilson disease [WD]). Values are expressed as Z-scores to enable direct comparison between markers. ExcCu (steel blue) and REC (green) show progressively higher levels from control to AD to WD. ExcCu is particularly elevated in AD and WD, while REC provides greater separation between AD and WD. This visualization highlights the distinct diagnostic profiles of the two copper-based biomarkers.

A one-way ANOVA revealed a significant difference in REC values among the three diagnostic groups, F(2,223) = 96.83, *p* < 0.0001. Post hoc Tukey tests showed that REC levels were significantly higher in AD vs. controls (mean difference = 2.33%, *p* < 0.001), WD vs. controls (mean difference = 7.76%, *p* < 0.001), and WD vs. AD (mean difference = 5.43%, *p* < 0.001). Standardized values (Z-scores) of ExcCu and REC showed progressive increases from controls to AD to WD. ExcCu was particularly elevated in AD and WD, while REC more distinctly separated AD from WD, highlighting their complementary diagnostic profiles (Figure 6).

ROC curve analysis demonstrated the good diagnostic performance of REC in differentiating the groups. The AUC was 0.68 for AD vs. controls, 0.92 for WD vs. controls, and 0.88 for WD vs. AD, indicating particularly high discriminative ability in identifying WD.

## 4. Discussion

The main result of this study is the validation of a direct ICP-MS-based assay for ExcCu, developed in compliance with CLSI standards. The method demonstrated excellent analytical precision, with ExcCu identifying AD patients with copper-related dysregulation at a specificity of 94.2%. This finding is particularly relevant given the modest cognitive benefits of currently approved anti-amyloid therapies [1,2,3] and the increasing recognition that AD likely encompasses multiple biologically distinct subtypes. Current diagnostic frameworks based on Aβ, tau, and neuroimaging may not fully reflect this heterogeneity. ExcCu addresses this gap by capturing copper-related alterations, potentially linked to oxidative stress and ATP7B dysfunction [35,36,37,38]. The validation of this method, which provides a direct quantification via ICP-MS, represents a methodological advancement over previous approaches [22,23,39,40,41,42], including the fluorescent-based assay developed by our group in 2017 [17], which was limited by signal instability and logistical constraints. Unlike the calculated non-Cp Cu, ExcCu avoids analytical propagation errors and physiologically implausible negative values [16].

Our current findings confirm significantly elevated ExcCu levels in AD patients compared to healthy controls. Moreover, the inclusion of WD patients—a condition marked by severe copper dysregulation—allowed for a broader evaluation of non-Cp Cu behavior. Both AD and WD groups exhibited increased non-Cp Cu, reinforcing shared pathophysiological features. The refined reference interval for ExcCu in healthy subjects (0.27–1.90 µmol/L), derived from a robust control cohort (n = 154), adds further value for clinical application.

Bland–Altman analysis showed limited agreement between the calculated non-Cp Cu and ExcCu, with persistent bias even after excluding negative values. This supports the view that while the two methods target the same biological pool, ExcCu offers superior analytical performance and better reflects pathophysiological changes at higher copper levels. The moderate correlation and frequent disagreement between ExcCu and non-Cp Cu observed in this study are consistent with prior findings and reflect the methodological limitations of Walshe’s formula. As extensively discussed elsewhere [17], the formula relies on key assumptions: the full saturation of ceruloplasmin with copper, analytical concordance between ceruloplasmin and copper concentrations (as reflected by the Cu:Cp ratio), and the negligible contribution of copper bound to other serum proteins. These conditions are often unmet in clinical practice. Moreover, ceruloplasmin measurements—particularly immunologic assays—are prone to overestimation and inter-laboratory variability, leading to physiologically implausible negative values of non-Cp Cu. Although the Cu:Cp ratio has been proposed as an internal consistency check [27,43], it is itself affected by the lack of assay standardization [43]. In contrast, ExcCu is a directly quantified parameter and is not subject to these propagation errors, providing a more robust reflection of bioavailable copper.

Among copper biomarkers, ExcCu and the relative exchangeable copper index (REC) demonstrated distinct diagnostic profiles. ExcCu was more effective in distinguishing AD from healthy controls (AUC = 0.81), while REC better discriminated AD from WD (AUC = 0.85), consistent with prior studies [29,30]. Although both reflect the labile copper pool, ExcCu appears more sensitive to the moderate copper dysregulation typical of AD, whereas REC is more specific for the severe disturbances found in WD.

Measuring ExcCu in AD is potentially useful in light of cuproptosis. Redox-active-ExcCu, elevated in many AD patients, can cross the blood–brain barrier (BBB), contributing to oxidative stress and mitochondrial dysfunction, both hallmarks of AD. Cuproptosis is triggered by excess intracellular copper binding to lipoylated mitochondrial proteins within the pyruvate dehydrogenase complex, leading to protein aggregation and cell death. As neurons are highly dependent on mitochondrial function, they are especially vulnerable.

Although there are still few experimental and system biology studies that link cuproptosis and AD through Ferredoxin 1 (FDX1, a key cuproptosis gene) and *APOE* ε4/ε4 [44,45,46,47,48,49,50], the mechanistic overlap is compelling.

FDX1 demonstrated significantly higher expression in peripheral blood and neuron models of AD than in non-AD individuals, with significantly higher expression in the APOE ε4/ε4 genotype than other APOE genotypes of AD patients [44]. It may promote cuproptosis in AD neurons by regulating the lipidation levels of the dihydrolipoamide S-acetyltransferase (DLAT) and dihydrolipoamide S-succinyl transferase (DLST) genes of the TCA cycle, thereby participating in the onset and development of AD. Therefore, ExcCu could serve as a proxy for neuronal copper load, which may be tipping cells toward cuproptotic death. This hypothesis is further reinforced by clinical and genetic parallels between AD and WD, the prototypical disorder of copper homeostasis [8]. In our previous meta-analysis [10], we demonstrated that AD is characterized by a paradoxical copper imbalance: reduced copper levels in the brain parenchyma, alongside increased labile (i.e., non–ceruloplasmin-bound in the brain) and non-Cp Cu in serum and interstitial fluids. This resembles the copper misdistribution seen in WD, where *ATP7B* mutations lead to intracellular hepatic copper depletion and extracellular accumulation. Notably, *ATP7B* has also been identified as a genetic risk factor for AD in patients with high serum labile copper [10,51]. In light of recent findings by Tsvetkov et al. [15], showing that Atp7b knockout in mice triggers copper accumulation, protein lipoylation dysfunction, and cuproptosis, we propose that a similar mechanism may underlie a copper-related subtype of AD [52]. In this model, impaired *ATP7B* function may lead to focal intracellular copper accumulation in neurons, tipping them toward cuproptotic cell death despite global copper deficiency—a mechanistic link that warrants further investigation.

In summary, ExcCu represents a reliable biomarker for copper-related phenotypes in AD and may serve as a useful stratification tool in future therapeutic trials/personalized interventions. The combination of methodological rigor, clinical validation, and the inclusion of a broad diagnostic spectrum reinforces its potential utility in neurodegenerative disorders linked to copper metabolism.

## 5. Conclusions

The ExcCu test, based on ICP-MS and validated under CLSI protocols, demonstrates excellent precision, especially at higher copper concentrations, linearity across the tested concentration range, a low detection limit suitable for clinical applications, and high recovery rates, confirming accuracy. This method is suitable for clinical diagnostics and research, particularly in disorders related to copper dysregulation.

## Figures and Tables

**Table 1:** Analytical precision of exchangeable copper (ExcCu) at low, medium, and high concentrations.

ExcCu Level (µmol/L)	Mean	Repeatability SD (CV%)	Within-Lab SD (CV%)	
Low (0.86)	0.862	0.035 (4.1%)	0.059 (6.8%)	
Medium (1.21)	1.209	0.048 (4.0%)	0.061 (5.0%)	
High (2.08)	2.080	0.056 (2.7%)	0.092 (4.4%)	
**(A) Low ExcCu level (0.86 µmol/L).**				
**Component**	**% of Total**	**SD**	**95% CI**	**CV**
Repeatability	36.0%	0.035	0.029 to 0.045	4.1%
Between Run	18.3%	0.025	0.000 to 0.045	2.9%
Within Day	54.3%	0.043	0.036 to 0.057	5.0%
Between Day	45.7%	0.040	0.021 to 0.064	4.6%
Within Laboratory	100.0%	0.059	0.050 to 0.078	6.8%
**(B) Medium ExcCu level (1.21 µmol/L).**				
**Component**	**% of Total**	**SD**	**95% CI**	**CV**
Repeatability	63.8%	0.048	0.040 to 0.062	4.0%
Between Run	14.0%	0.023	0.000 to 0.049	1.9%
Within Day	77.8%	0.053	0.045 to 0.069	4.4%
Between Day	22.2%	0.029	0.000 to 0.052	2.4%
Within Laboratory	100.0%	0.061	0.053 to 0.077	5.0%
**(C) High ExcCu level (2.08 µmol/L).**				
**Component**	**% of Total**	**SD**	**95% CI**	**CV**
Repeatability	37.8%	0.056	0.046 to 0.072	2.7%
Between Run	12.8%	0.033	0.000 to 0.063	1.6%
Within Day	50.6%	0.065	0.055 to 0.085	3.1%
Between Day	49.4%	0.064	0.038 to 0.102	3.1%
Within Laboratory	100.0%	0.092	0.077 to 0.122	4.4%

The 95% confidence intervals were calculated using Exact or Maximum Likelihood Statistics (MLS) methods, according to CLSI EP05-A3 guidelines.

**Table 2:** Regression model parameters for exchangeable copper (ExcCu).

**Linear Fit**					
**Parameter**	**Estimate**	**SE**	**t**	**DF**	***p*-Value**
Constant	0.3255	0.14239	2.29	16	0.0362
X	0.05775	0.0036715	−256.64	16	<0.0001
**2nd-Order Polynomial Fit**					
**Parameter**	**Estimate**	**SE**	**t**	**DF**	***p*-Value**
Constant	0.4129	0.78564	0.53	15	0.6069
X	0.05295	0.04255	−22.26	15	<0.0001
X^2^	6.313 × 10^−5^	0.00055764	0.11	15	0.9114
**3rd-Order Polynomial Fit**					
**Parameter**	**Estimate**	**SE**	**t**	**DF**	***p*-Value**
Constant	3.155	4.5581	0.69	14	0.5002
X	−0.1750	0.3755	−3.13	14	0.0074
X^2^	0.006212	0.010078	0.62	14	0.5475
X^3^	−5.393 × 10^−5^	8.8262 × 10^−5^	−0.61	14	0.5509

Linear Fit: Root Mean Square Error (RMSE): 0.121; 2nd-Order Polynomial Fit: RMSE: 0.125; 3rd-Order Polynomial Fit: RMSE: 0.127; RMSE quantifies the average deviation between observed values and those predicted by the model. A lower RMSE value indicates a better model fit to the data. In this table, the linear model shows the lowest RMSE, suggesting that it provides the best predictive accuracy among the three models evaluated.

**Table 3:** Determination of detection limits for exchangeable copper (ExcCu) (LoB and LoD).

**Limit of Blank (LoB)**				
**N**	**Mean**	**SD**	**Alpha**	**Critical Value (LoB)**
60	0.00010	0.00008	5%	0.0002
**Limit of Detection (LoD)**				
**N**	**Pooled SD**	**Beta**	**Detection Limit (LoD)**	
60	0.02387	5%	0.0397	

Limit of Blank (LoB): Material: Blank; Limit of Detection (LoD): Material: Non-blank.

## Data Availability

The original contributions presented in this study are included in the article/Supplementary Materials. Further inquiries can be directed to the corresponding author(s).

## Supplementary Materials

The following supporting information can be downloaded at: https://www.mdpi.com/article/10.3390/biom15060788/s1, Table S1. ICP-MS Settings for Copper Quantification; Table S2: Concentration levels and relative fits for exchangeable copper (ExcCu); Table S3. Recovery rates from spiked serum samples.
