# Supplementary material for: CLSI Validation of Exchangeable Copper Determination in Serum by ICP-MS: A Focus on Alzheimer’s Disease and Wilson Disease
Source: Biomolecules. 2025 May 29;15(6):788. doi: 10.3390/biom15060788 (PMC12190365; doi:10.3390/biom15060788)
Supplement: Supplementary file 1 [file biomolecules-15-00788-s001.zip › biomolecules-3611940-supplementary.pdf]

Supplementary Materials

Table S1 – ICP-MS Settings for Copper Quantification

Parameter	Setting
**Instrument model**	iCAP Q ICP-MS (ThermoFisher Scientific)
**Isotopes monitored**	^{63}Cu, ^{65}Cu
**RF Power**	1550 W
**Plasma gas flow (Ar)**	15.0 L/min
**Auxiliary gas flow**	0.9 L/min
**Carrier gas flow (nebulizer Ar)**	1.0 L/min
**Nebulizer**	PFA concentric
**Spray chamber**	Cyclonic, cooled to 2–4°C
**Cones**	Nickel sampler and skimmer cones
**Sample introduction rate**	0.4–0.6 mL/min
**Dwell time per isotope**	100 ms
**Replicates per sample**	3–5
Internal standard	^{89}Y (Yttrium, 100 µg/L in 2% HNO_3)
Calibration range	1, 10, 100 µg/L (prepared from 10000 µg/L stock)
Diluent	2% HNO_3 containing internal standard (Y)

Table S2: Concentration levels and relative fits for exchangeable copper (ExcCu).

Dilution	Linear fit	Nonlinear fit	Nonlinearity	95% CI	Relative nonlinearity
26	1.827	-	-	- to -	-
29	2.000	-	-	- to -	-
32	2.174	-	-	- to -	-
35	2.347	-	-	- to -	-
38	2.520	-	-	- to -	-
41	2.693	-	-	- to -	-
44	2.867	-	-	- to -	-
47	3.040	-	-	- to -	-
50	3.213	-	-	- to -	-

No 2nd/3rd order polynomial fit is statistically better than a linear fit at the 5% significance level.

Table S3. Recovery rates from spiked serum samples

	Recovery %
**standard ExcCu serum+ 0.5 microM**	96
**standard ExcCu serum+ 1 microM**	95
**standard ExcCu serum+ 2 microM**	95
**standard ExcCu serum+ 3 microM**	94.6

**standard ExcCu serum+ 5 microM**	95
**mean value**	95
